# Is there equity in initial access to formal dementia care in Europe? The Andersen Model applied to the Actifcare cohort

**DOI:** 10.1002/gps.5213

**Published:** 2019-11-06

**Authors:** Liselot Kerpershoek, Marjolein de Vugt, Claire Wolfs, Martin Orrell, Bob Woods, Hannah Jelley, Gabriele Meyer, Anja Bieber, Astrid Stephan, Geir Selbæk, Mona Michelet, Anders Wimo, Ron Handels, Kate Irving, Louise Hopper, Manuel Gonçalves‐Pereira, Conceição Balsinha, Orazio Zanetti, Daniel Portolani, Frans Verhey, the Actifcare Consortium

**Affiliations:** ^1^ Maastricht University Alzheimer Centrum Limburg NL The Netherlands; ^2^ Nottingham University Institute of Mental Health Nottingham UK; ^3^ Bangor University UK; ^4^ Martin‐Luther University Halle‐Wittenberg DE; ^5^ Norwegian National Advisory Unit on Ageing and Health Vestfold Hospital Tønsberg Norway; ^6^ Dept of Geriatric Medicine Oslo University Hospital Oslo Norway; ^7^ Faculty of medicine University of Oslo Oslo Norway NO; ^8^ Department of Neurobiology, Care sciences and Society Karolinska Institutet Stockholm SE; ^9^ School of Nursing, Psychotherapy and Community Health Dublin City University IE; ^10^ CEDOC, Nova Medical School|Faculdade de Ciências Médicas Universidade Nova de Lisboa PT; ^11^ IRCSS Istituto Centro S. Giovanni di Dio Fatebenefratelli Brescia IT

**Keywords:** access to care, Andersen model, equity, middle‐stage dementia, service use

## Abstract

**Objectives:**

In the current study, the Anderson model is used to determine equitable access to dementia care in Europe. Predisposing, enabling, and need variables were investigated to find out whether there is equitable access to dementia‐specific formal care services. Results can identify which specific factors should be a target to improve access.

**Methods:**

A total of 451 People with middle‐stage dementia and their informal carers from eight European countries were included. At baseline, there was no use of formal care yet, but people were expected to start using formal care within the next year. Logistic regressions were carried out with one of four clusters of service use as dependent variables (home social care, home personal care, day care, admission). The independent variables (predisposing, enabling, and need variables) were added to the regression in blocks.

**Results:**

The most significant predictors for the different care clusters are disease severity, a higher sum of (un)met needs, hours spent on informal care, living alone, age, region of residence, and gender.

**Conclusion:**

The Andersen model provided for this cohort the insight that (besides need factors) the predisposing variables region of residence, gender, and age do play a role in finding access to care. In addition, it showed us that the numbers of hours spent on informal care, living alone, needs, and disease severity are also important predictors within the model's framework. Health care professionals should pay attention to these predisposing factors to ensure that they do not become barriers for those in need for care.

**Key Points**

- The Andersen model provided the insight that (besides need factors)the predisposing variables region of residence, gender and age do play a role in finding access to care.
- Number of hours spent on informal care, living alone, needs and disease severity are important predictors for service use.
- Healthcare professionals should pay attention to these predisposing factors to ensure that they don't become barriers for those in need for care.

## 1 INTRODUCTION

Dementia has a major influence on a person's life, affecting not only cognitive abilities, but also activities of daily living. As the condition progresses, an increasing amount of care and support is needed, typically commencing with care from family and friends,^1^ often described as “informal care.” When informal care alone may not suffice, care from health and social care agencies (“formal care”) in the community may be required and might postpone the need for institutionalization. For example, the use of in‐home help (personal care and companionship) early in the dementia process may delay nursing home placement.^2^

A literature review showed that one third of informal carers of people with dementia does not make use of community services.^3^ Reasons for nonuse of services vary from the consideration that services are not needed, to a lack of awareness or refusal of the care recipient. In a recent review, Phillipson et al describe that the majority of people with dementia and their informal carers experience difficulties in finding access to formal care services. When care is received, often it is not of the right type.^4^ It is widely accepted across many health care systems that there should be equity of access to services^5^^.^ 6 Equity can be explained as the absence of systematic discrepancies in access to care services, with equal access for those with equal needs. Possible reasons for inequality arise from differences in availability, quality, costs, and information for different population groups.^7^ A recent research project encompassing the Netherlands, Germany, Italy, Belgium, Finland, and Iceland (Assessing Needs of Care In European Nations) reported that there is low equity in access to home health care services for older people in Italy and Finland.^8^ When looking at overall health care for older people in Europe, approximately 50% to 75% of all formal long‐term care is delivered at home.^8^ There are major differences in how care is subsidized, organized, and delivered. This is similar for dementia‐specific care. The recent “European Dementia Monitor” showed differences on a financial, organizational, and practical level across Europe regarding access to dementia care and treatment leading to inequality.^9^ Reports from WHO^10^ and Alzheimer's Disease International (ADI) have shown a great and unequal distribution of dementia care resources worldwide.^11^

This emphasizes the importance of identifying factors that can facilitate or hamper access to formal care. A model that considers this mix of factors is the Andersen Behavioural Model of Health Service Use.^12^ The model identifies predisposing, enabling factors, and need factors to explain service use, and can indicate inequity in access to care. Predisposing factors include demographic information, such as age or level of education. Enabling factors include variables that either facilitate or hamper access to care such as travelling distance to a care facility or waiting lists. Need factors express the perceived and evaluated needs of persons, based on their mental and physical condition.^12^ Equitable access is said to occur for patients when need and enabling factors such as disease severity and waiting lists determine realized access (actual use of formal care). Inequitable access is said to occur for patients if predisposing factors such as gender, social economic status, or education contribute significantly to use of formal care, after controlling for need and enabling factors.

In the current European Actifcare study, access to care is studied using the Andersen model. People with dementia and their informal carers from eight European countries (the Netherlands, Germany, United Kingdom, Ireland, Sweden, Norway, Italy, and Portugal) were followed for 1 year during which a transition to formal care was considered likely. By means of a set of questionnaires, a number of predisposing, enabling, and need predictors of formal care use was mapped.^13^ We aimed at investigating these predictors to find out whether there is equitable access to dementia‐specific formal care services in Europe. Consequently, results can identify which specific factors should be a target for interventions to improve equitable access.

## 2 METHODS

### 2.1 Study design and participants

Study data were collected as part of a European prospective cohort study: Access to Timely Formal Care (Actifcare). The design has been described elsewhere in detail^13^^.^ 14 In total, 451 dyads of community‐dwelling people with mild to moderate dementia who were expected to use formal care within 1 year and their informal carers were recruited via memory clinics, general practices, casemanagers, and community mental health teams. The expected use of care was based on clinical judgment by an experienced clinician. At baseline, the participants had yet to use formal care support, involving personal care from a paid worker, in relation to the dementia. In the Actifcare cohort study, formal care was operationalized as home nursing care, day care services, community or long‐term medical care, and social care structures. It did not include day care received solely for social purposes, domestic home help, housekeepers, volunteers, support groups, transport services, and meal programs. Written informed consent was obtained from both the person with dementia and the carer according to the national procedure in each country. All countries obtained the appropriate ethical approvals.^13^

### 2.2 Measurements

Measurements were scheduled at baseline (T0), six (T1), and twelve (T2) months. Comprehensive assessments were conducted by two researchers, who in the majority of the cases visited the dyad at home. Details are described elsewhere.^13^ The variables in our analyses were divided into three groups according to the Andersen Model: predisposing, enabling, and need variables.

#### 2.2.1 Predisposing variables

For both the informal carer and the care recipient, demographic characteristics recorded were age, gender, social economic status, and years of education. For both members of the dyad, these characteristics were analysed separately. Social economic status was generated by an occupation coding system with five classes (1 = Higher managerial, administrative and professional occupations, 2 = Intermediate occupations, 3 = Small employers and own account workers, 4 = Lower supervisory and technical occupations, 5 = Semi‐routine and routine occupations).^15^ In addition, region of residence was reported, where Norway and Sweden were classified as “North,” while Ireland, United Kingdom, Germany, and the Netherlands were “Middle” regions. Portugal and Italy were classified as “South.” This classification was based on similarities in health care provision and culture. In addition, this division was made based on previous (qualitative) outcomes of the Actifcare project.

#### 2.2.2 Enabling variables

Informal care was reflected by the number of informal carers and the number of hours spent on informal care by the main carer. Both these variables were derived from the Resource Utilization in Dementia scale (RUD),^16^ which is designed to measure informal and formal care use. An important demographic variable that may relate to service use is living situation. Several studies have shown that if a person with dementia lives together with their carer(s), they are less likely to use services.^17^, ^18^

#### 2.2.3 Need variables

##### 2.2.3.1 Disease severity

To measure disease severity, two variables were used: severity of dementia impairment and daily functioning. Severity of dementia impairment was measured with the researcher‐rated Clinical Dementia Rating (CDR),^19^ where we used the sum of boxes instead of the total score, since the sum of boxes gives a more fine‐grained representation of cognition and functioning, and it allows to monitor change between follow‐ups.^20^ In addition, the Mini Mental State Examination (MMSE) scale was used, which is a scale with a range from 0 to 30, where lower scores indicate higher cognitive impairment.^21^ To measure daily functioning, the Instrumental Activities of Daily Life (IADL) scale and Physical Self Maintenance Scale (PSMS)^22^ were used. The IADL scale consists of eight items providing information about performance on daily activities such as shopping and handling money. The six‐item PSMS focuses on self‐care abilities such as washing and walking. For both scales, a higher score indicates worse functioning.

##### 2.2.3.2 Needs

Needs were assessed by the Camberwell Assessment of Need for the Elderly (CANE), which is a semi‐structured interview tool concerning (un)met needs in 24 medical, psychological, and social areas. In this study, the scale was rated using both the perspective of the person with dementia and the informal carer, as well as the interpretation by the researcher while keeping in mind the different opinions that were gathered. A need was considered met if it is provided for, either by informal or formal care.^23^ In this study, we used the number of met and the number of unmet needs rated by the researcher.

##### 2.2.3.3 Behavioural problems

Behavioural problems of the person with dementia were assessed with the short version of the Neuropsychiatric Inventory Questionnaire (NPI‐Q), which is a structured interview tool carried out by the researcher interviewing the informal carer. Information on 12 neuropsychiatric symptoms was gathered; delusions, hallucinations, agitation/aggression, dysphoria, anxiety, euphoria, apathy, disinhibition, irritability/lability, aberrant motor behaviour, night time behaviour disturbances, and eating abnormalities. Higher scores indicate the presence of more behavioural symptoms.

#### 2.2.4 Service use

Service use was administered with a specifically for this study constructed service use checklist, which consisted of 22 to 26 items (depending on availability per country). Usage of each service was recorded at each assessment point. Four clusters of formal care services were created in which a distinction was made between services which provided support for the person with dementia and carer at home, but which were focused on providing company and activity (social activities), and those services providing personal care, eg, help with washing and dressing for the person with dementia at home. The clusters were (a) help at home (social), (b) help at home (personal care), (c) day care, and (d) admission to nursing home, care home, long‐term admission to hospital due to dementia. Next, we dichotomized usage on each cluster as positive if any of the services within a cluster at any of the two follow‐up time points was utilized.

#### 2.2.5 Ethics approval and consent to participate

All individual countries have applied for medical ethical approval in their own country. Ethical consideration differs between countries: Medische‐ ethische toetsingscommissie (NL), Wales Research Ethics Committee 5, Bangor (UK), Ethics committee of the Medical Faculty, Martin Luther University Halle‐Wittenberg (DE), Regional committee for medical and health research ethics, South‐East B (NO), the Regional Ethics Review Board (SW), Dublin City University Research Ethics Committee (IE), Ethics Committee of the Nova Medical School, Ethics Committee of Centro Hospitalar de Lisboa Ocidental, Ethics Committee of ARSLVT, Ethics Committee of ARSA, Comissão Nacional de Protecção de Dados (PT), Comitato Etico, and IRCCS San Giovanni di Dio‐ Fatebenefratelli (IT). All participating NHS sites in the UK received permission to perform the study. The carer and the person with dementia both signed a separate informed consent form, after they had sufficient time to read the form and ask questions if needed. The study protocol complies with the Medical Research Involving Human Subjects Act and codes on “good use” of clinical data.

### 2.3 Statistical analyses

Group characteristics were described using descriptive statistics. Logistic regression analyses were carried out with each of the four clusters of service use as dependent variable (home social care, home personal care, day care, admission). The independent variables were added to the regression in blocks: firstly, a block with all needs variables, secondly a block with enabling variables, and lastly a block with predisposing variables. This method is commonly used in analyses based on the Andersen model.^12^ Within each block, backward regression was used to keep all variables with p < =0.10, before continuing with the next block. SPSS version 24 was used to perform the analyses.

## 3 RESULTS

A total of 451 dyads participated in the study. The group characteristics are summarized in Table 1. Amongst the people with dementia, 48% were diagnosed with Alzheimer's Disease. The majority (78%) had a CDR stage score of 1 (mild dementia). The relation between the person with dementia and the informal carer was mainly spousal (60%), a parent‐child relation (30%), or other (10%) such as other family members or friends. A minority of 127 people with dementia (28%) lives alone. The percentages of service use uptake can be found in Table 2. Predictors of home social care at T1 or T2 are presented in Table 3. A higher CDR score, a higher sum of met needs, more hours spent on informal care at baseline, and living alone at baseline significantly predicted the use of home social care at T1 or T2. None of the predisposing variables added significantly to the prediction.

**Table 1:** Characteristics at baseline grouped according to Andersen's behavioural model (n = 451)

Predisposing Variables	
PwD male (n, %)	207 (46)
PwD female (n,%)	244 (54)
PwD age (mean, SD) *Range*	77.8 (7.9) [47‐92]
PwD education (mean years, SD)	9.8 (4.5)
PwD social economic status (n, %)	Class 1: 109(24)Class 2: 62 (14)Class 3: 48 (11)Class 4: 28 (6)Class 5: 126 (28)Missing: 78 (17)
Dementia type DSM‐IV (n, %)	
*Alzheimer's disease*	218 (48)
*Vascular dementia*	52 (11)
*Mixed dementia*	56 (12)
*Lewy body dementia*	6 (1)
*Unknown/Other*	117 (27)
*Missing*	2 (1)
IC male (n,%)	151 (33)
IC female (n,%)	300 (67)
IC age (mean, SD) *Range*	66.4 (13.2) [25‐92]
IC education (mean years, SD)	11.9 (4.4)
IC social economic status (n, %)	Class 1: 99 (22)Class 2: 60 (13)Class 3: 41 (9)Class 4: 6 (1)Class 5: 55 (12)Missing: 190 (43)
Region (n, %)	
*North*	110 (24)
*Middle*	222 (48)
*South*	119 (26)
Enabling factors	
Living alone (n, %)	127 (28)
Number of informal carers (mean, SD)	1.1 (1.2)
Hours of informal care per month (mean, SD, range)	98.9 (93.2) 0‐570
Need factors	
CDR sum of boxes (mean, range)	7.1 (2‐16)
MMSE score 0‐30 (mean, SD)	19 (4.9)
CANE unmet needs (mean, range)	1.87 (0‐17)
CANE met needs (mean, range)	8.2 (0‐19)
NPI (mean, range)	7.8 (0‐30)

CANE, Camberwell assessment of need for the elderly; CDR, clinical dementia rating; IC, informal carer; NPI, Neuropsychiatric Inventory; Pwd, person with dementia.

**Table 2:** Numbers and percentages of service use uptake for each care cluster

	Service Use at Either Follow‐Up
Home social care	33 (8.3%)
Home personal care	51 (11.3%)
Day care	42 (9.3%)
Admission	50 (11.1%)

**Table 3:** Significant baseline predictors of home social care at T1 or T2 in backward logistic regression

Block	Variable	B
Needs	CDR	1.13 (1.01‐1.27)**
	Met needs	1.12 (1.02‐1.23)**
Enabling	Living together	0.44 (0.23‐0.85)**
	IC hours	1.01 (1.00‐1.01)*

CDR, clinical dementia rating; IC, informal carer. Odd's ratio (lower CI‐upper CI 95%)

** *P* value <.05,

* *P* value < .01.

Predictors of home personal care at T1 or T2 are presented in Table 4. A higher CDR score, a higher sum of unmet needs, more hours spent on informal care at baseline, and living alone at baseline significantly predicted the use of home personal care at T1 or T2. The predisposing variable age of the person with dementia was significantly associated with service uptake in a positive direction. In addition, living in the North of Europe is significantly associated with the use of home personal care, in comparison to living in the South.

**Table 4:** Significant predictors of home personal care at T1 or T2 in backward logistic regression

Block	Variable	B
Needs	CDR	1.16 (1.04‐1.29)**
	Unmet needs	1.25 (1.09‐1.42)*
Enabling	Living together	0.29 (0.16‐0.52)*
	IC hours	1.00(1.00‐1.01)**
Predisposing	Pwd age	1.07 (1.00‐1.14)**
	Region South vs North	0.13 (0.03‐0.66)**

CDR, clinical dementia rating; IC, informal carer; Pwd, person with dementia. Odd's ratio (lower CI‐upper CI 95%)

** *P* value <.05,

* *P* value <.01.

For day care, a higher sum of met needs, living alone at baseline, and being a female informal carer predict use at T1 or T2 (see Table 5). In addition, living in the North of Europe is significantly associated with the use of day care, in comparison to living in the South or the Middle.

**Table 5:** Significant predictors of day care at T1 or T2 in backward logistic regression

Block	Variable	B
Needs	Met needs	1.13 (1.05‐1.22)**
Enabling	Living together	0.51 (0.30‐0.87)**
Predisposing	Gender IC (being female)	5.1 (1.27‐20.79)**
	Region South vs North	0.06 (0.01‐0.30)*
	Region Middle vs North	0.53 (0.16‐0.77)**

IC, informal carer. Odd's ratio (lower CI‐upper CI 95%)

** *P* value <.05,

* *P* value <.01.

As shown in Table 6, a higher CDR score, more hours spent on informal care at baseline, and being a male person with dementia significantly predict admission to a nursing home, care home, long‐term admission to hospital due to dementia at T1 or T2. Living in the North of Europe is significantly associated with admission, in comparison to living in the South or the Middle.

**Table 6:** Significant predictors of admission to a care home/nursing home at T1 or T2 in backward logistic regression

Block	Variable	B
Needs	CDR	1.25 (1.12‐1.41)*
Enabling	IC hours	1.00 (1.00‐1.01)**
Predisposing	Gender pwd (being female)	0.17 (0.04‐0.70)**
	Region South vs North	0.18 (0.04‐0.91)**
	Region Middle vs North	0.37 (0.16‐0.89)**

CDR, clinical dementia rating; IC, informal carer; Pwd, person with dementia. Odd's ratio (lower CI‐upper CI 95%)

** *P* value <.05,

* *P* value <.01.

## 4 DISCUSSION

This study examines factors associated with access to formal care for people with dementia and their informal carers, based on the Andersen's framework, which can be used to determine equity regarding access to care.

According to the Andersen model, access to care is considered equitable if it is predicted by enabling and need factors, and not by predisposing factors.^12^ In our cohort, the major predictors for service use were indeed need factors, namely met or unmet needs as measured by the CANE and disease severity reflected by a higher CDR sum of boxes. This is an indication for equity in access to care.

There were also enabling factors predicting care use in most of the clusters. The first one was a higher number of hours spent by the informal caregiver on care tasks. There are complex interactions between informal and formal care, and studies on this subject are scarce.^24^ Previous studies have shown that spousal caregivers often express reluctance to start using formal care because they perceive their tasks as a moral obligation.^25^ Previous studies have found comparable results, with associations between a higher amount of caregiving hours and subsequent institutionalisation.^26^ A logical explanation here could be that more hours spent indicates a necessity for more care due to disease severity. This seems to be in line with a review investigating the burden of caregivers of people with dementia, which reported that objective burden significantly increases with growing disease severity.^27^

The enabling factor “living alone” predicted three subtypes of care (home social care, home personal care, and day care), which is in line with previous research,^28^ where people with dementia who were living alone were more likely to receive home help with everyday tasks and meals on wheels. In absence of an informal caregiver living with the person with dementia, the need for help might be more urgent. “Living alone” was not a predictor for admission due to dementia in our cohort, which is not in line with previous studies.^29^, ^30^ A possible explanation for this is that the percentage of people who were admitted to a nursing/care home in our cohort is rather low, since the follow‐up period was only one year.

There were also predisposing factors significantly predicting access to subtypes of care. A higher age of the person with dementia was related to home personal care uptake. This might partly be explained by the fact that frailty increases with age and dependence and the need for support with self‐care increases accordingly.^31^ Although we asked about services related to the person's dementia, the complexity of interactions between cognitive and physical impairment makes it difficult to delineate services that are intended to meet needs arising from comorbid physical health problems from those arising from dementia.

Day care was started earlier where there was a female primary caregiver, and admission was used earlier when the person with dementia was male. Our results are consistent with a systematic review by Luppa et al which found that being a male person with dementia is a predictor for institutionalization.^26^ However, in a different review no significant gender differences were found.^29^ It is therefore difficult to find an explanation for these results without taking into account the whole range of variables that may differ between studies.

As the Andersen model states, it is undesirable that predisposing factors predict care use in people with identical care needs, as this reflects a discrimination towards a person's demographic characteristics. Gender and age do seem to play a role in equitable access to care, but the explanatory mechanism for this deserves further attention. The predisposing factors education and social economic status were not hindering or facilitating access to dementia care in this cohort. This is not in line with recent findings, in which a clear difference between countries was established in (amongst others) availability and affordability of care. In a large study into medical care, inequity was found in nearly half of the Economic Cooperation and Development (OECD) countries. This was the case for visits to a physician, where richer patients found easier access to care, especially in Mexico, the US, Finland, Portugal, and Sweden.^32^ In addition, people with a higher social economic status were more likely to visit a specialist. The fact that we did not find inequity due to social economic status might be because we focused solely on dementia care and on countries that are all part of Western Europe. In addition, this finding could be partly explained by the relation between region and social economic status.

Lastly, we studied the effect of region of residence in this cohort. People with dementia who were living in the North of Europe find easier access to care, which is in line with previous findings. Recently, in “The European Dementia Monitor,” the highest ranked country was also a Northern European country, namely Finland. Shortly after Finland, the Middle European countries followed (United Kingdom, the Netherlands, and Germany).^9^ In a different study, Southern European countries had a 5.0‐fold lower chance of complementing informal with formal care. The authors suggested that culture (including the representation of family caregiving as a moral obligation) likely played a role in their results. In addition, they found that people from Denmark or the Netherlands have a 2.4‐fold greater chance of complementing informal with formal care in contrast to other Middle European countries.^25^ This might partly be explained by the larger availability of care in Northern versus Southern countries.^33^ Another factor that should be kept in mind in this context is that the division of countries into regions might be too simplified because there are substantial differences in the care systems of the participating countries in the middle region. Differences in individual financial contributions between countries are also likely to lead to differences in service use between countries. The inclusion of these factors went beyond the scope of this manuscript.

## 5 CONCLUSION

The Andersen model is useful, but the accompanying analyses bring along limitations. As with any model, it is a simplification of reality. The model does not predefine moderating or mediating effects, which, if correctly identified, could better reflect the potential underlying mechanism for care consumption. In future studies, additions can be made to the model, eg, in terms of additional factors or interactions in order to reveal the complex pathways of access to care. Due to restrictions caused by the prespecified categories of variables in the model, there is a large group of variables that are not considered here. However, these variables might influence access to care to a great extent, such as carer characteristics, subjective burden, and quality of life. The influence of these factors can be investigated in a separate article with analyses outside the framework of this model. Another limitation of this study is that, based on the inclusion criteria, people were expected to start using formal care within the next year. However, the numbers of service use uptake were smaller than expected, probably because the study period may have been too short. As we were limited by the current design of the study, the follow‐up period was extended with assessments at 36, 48, and 60 months to assess more closely where care transitions take place and which factors predict these transitions. In addition, it would have been informative to include information on income, as this is a better reflection of current social economic status than previous occupation. Although the sample was meant to be typical, generalization of the results to individual countries should be handled with caution as the cohort consisted of a convenience sample. For this cohort, the Andersen model provided the insight that besides need factors the predisposing factors region of residence, gender, and age do play a role in finding access to care. In addition, it showed us that more hours spent on informal care, living alone, needs, and disease severity are important predictors. Health care professionals should pay attention to these predisposing factors to ensure that these do not act as barriers for those in need for dementia care.

## DISCLOSURE STATEMENT

The authors have reported no conflicts of interest.

## FUNDING

The project is supported through the following funding organizations under the aegis of JPND—http://www.jpnd.eu [grant number 733051001]. Germany, Ministry of Education and Research, Ireland, Health research board, Italy, Ministry of Health, the Netherlands, The Netherlands organization for Health Research and Development, Sweden, The Swedish Research Council for Health, Working Life and Welfare, Norway, The Research Council of Norway, Portugal, Foundation for Science and Technology (Fundação para a Ciência e Tecnologia [grant number FCT‐JPND‐HC/0001/2012], United Kingdom, Economic and Social Research Council. JPND has read and approved of the protocol of the Actifcare study.

## Data Availability

The data that support the findings of this study are available from the corresponding author upon reasonable request.
